# Exploring the predictive role of personal identity in trait anxiety: network and Bayesian evidence from Chinese college students

**DOI:** 10.3389/fpsyg.2025.1459306

**Published:** 2025-05-02

**Authors:** Ruizhi Huang, Ye Yuan, Huiqing Shen, Yuqi Sun, Zhilin Wang, Huilin Qiu, Jaesik Jeong, Wenjing Yan, Ke Jiang

**Affiliations:** ^1^Teacher Education College, Lishui University, Lishui, Zhejiang, China; ^2^School of Mental Health, Wenzhou Medical University, Wenzhou, China; ^3^Department of Mathematics and Statistics, Chonnam National University, Gwangju, Republic of Korea; ^4^Zhejiang Provincial Clinical Research Center for Mental Disorders, The Affiliated Wenzhou Kangning Hospital, Wenzhou, China; ^5^Mental Health Education and Research Center, School of Marxism, Nanjing Medical University, Nanjing, China; ^6^English Department, College of Foreign Languages and Cultures, Sichuan University, Chengdu, China; ^7^The Affiliated Kangning Hospital of Wenzhou Medical University, Wenzhou, China; ^8^Center for Brain, Mind and Education, Shaoxing University, Shaoxing, China

**Keywords:** personal identity, self, trait anxiety, LASSO, Bayesian network model

## Abstract

**Introduction:**

Personal identity, defined as the perceived continuity and coherence of the self over time, has been theorized to influence anxiety. This study investigates whether personal identity predicts trait anxiety and examines the mediating roles of personality traits and meaning in life.

**Methods:**

A total of 613 Chinese college students completed standardized questionnaires, including the State-Trait Anxiety Inventory (STAI), Self-Identity Scale (SIS), Eysenck Personality Questionnaire (EPQ), and the Meaning in Life Questionnaire (MLQ). Data were analyzed using adaptive LASSO network analysis and Bayesian modeling.

**Results:**

Personal identity showed strong predictive power for trait anxiety in both network and Bayesian models. It demonstrated centrality in the anxiety-related psychological network, with high predictive accuracy in Bayesian classification (88.6%).

**Discussion:**

The findings suggest that a stable and coherent sense of personal identity may protect against anxiety. Conceptually, anxiety is interpreted as arising from disruptions in self-synthesis. These results provide evidence for identity-based approaches to understanding and managing anxiety in university student populations.

## 1 Introduction

### 1.1 Trait anxiety

Anxiety, a highly significant emotion, has been extensively examined by philosophers, writers, and psychologists (Camus, 1954; Starkstein, 2018), who recognize it as a critical area of research (Endler and Kocovski, 2001; Freud, 1936). As a symptom often indicative of poor mental health, anxiety frequently co-occurs with various other mental health conditions. It is recognized as a primary or fundamental symptom in numerous psychological disorders (Pallanti et al., 2013; Wu et al., 2018; Baker et al., 2020). Anxiety is a complex psychological response to perceived threats or uncertainties, typically characterized by feelings of fear, tension, and unease (American Psychiatric Association, 2013). It is commonly conceptualized in two forms: state anxiety, a transient emotional reaction to situational stressors, and trait anxiety, a stable individual difference reflecting a predisposition to perceive situations as threatening (Spielberger, 1966; Cattell and Scheier, 1958). This distinction is well-established and supported by extensive empirical research (Endler and Kocovski, 2001). While anxiety frequently co-occurs with various psychological conditions, a formal diagnosis of an anxiety disorder requires clinical evaluation and treatment. In the general population, the one-year prevalence of anxiety disorders ranges from 10 to 14%, rising to 20–30% among adolescents aged 15–25 (Craske and Stein, 2016). On a global scale, anxiety disorders contribute significantly to the disease burden, accounting for 3.3% of the total and incurring societal costs estimated at €74 billion across 30 European countries (Gustavsson et al., 2011). In China, anxiety disorders are the most prevalent class of mental disorders, with a 12-month weighted prevalence of 5.0% (4.2–5.8) and a lifetime prevalence of 7.6% (6.3–8.8) (Huang et al., 2019).

### 1.2 Personal identity and its relationship with anxiety

A growing body of research has examined the complex relationship between personal identity and anxiety, though findings remain inconclusive (Hirsh and Kang, 2016; Hayward et al., 2020), individuals experiencing more anxiety with identity moratorium (Lillevoll et al., 2013; Marcia, 1976). Fluctuations in personal identity have also been shown to predict short-term changes in anxiety and depression (Schwartz et al., 2011), likely due to the impact of identity states on the perception and processing of environmental stimuli. Additionally, there is evidence that anxiety and other mental health issues can influence personal identity (Marcotte and Lévesque, 2018; Campbell, 2018), possibly as emotional states induce cognitive biases affecting self-concept synthesis. The failure to reconcile different self-aspects into a coherent identity can cause severe identity distress, correlating with mental health challenges like anxiety (Berman, 2019).

Classic psychological theories offer further insight into this bidirectional relationship. Erik Erikson, a pioneer in studying personal identity, conceptualized ego identity as comprising four interactive aspects: (1) self-sameness and recognition of one’s unique characteristics; (2) social validation through interactions with others; (3) roles exploration for purpose formation; and (4) dynamic integration of experiences over time (Erikson, 1956). Marcia expanded on Erikson’s theory by defining four identity status categories, proposing that varying levels of exploration and commitment result in different outcomes in resolving identity crises (Marcia, 1966). These frameworks emphasize identity as a dynamic and multifaceted construct—a view that supports its theoretical linkage to emotional outcomes such as anxiety. In addition to psychological theories, philosophical perspectives have also contributed to the conceptualization of personal identity. Kant’s view of consciousness, for instance, has had a profound impact on the field (Brook, 2003; Longuenesse, 2013). Contemporary scholars argue that personal identity must be understood through both the structural conditions of consciousness and the lived continuity of experiences (Drummond, 2020). Notably, the psychological concept of personal identity stems from philosophical investigations into Numerical Identity—the enduring uniqueness of entities over time, emphasizing each entity’s relationship with itself (Morrison, 2022; Noonan and Curtis, 2022; Olson, 2022).

For the purposes of this study, we adopt a definition of personal identity grounded in both Eriksonian theory and the concept of numerical identity. Specifically, personal identity is defined as the unique, enduring continuity of the self over time, involving both psychological coherence and self-recognition. This conceptualization aligns with Erikson’s emphasis on self-sameness, societal validation, and temporal integration. Based on this framework, we hypothesize that personal identity may serve as a predictor of trait anxiety. While trait anxiety reflects stable behavioral tendencies in response to perceived threats, personal identity represents a core structural feature of the individual self that may shape one’s emotional resilience and vulnerability.

The connection between personality traits and anxiety has long been a central focus in psychological research. Personality is commonly defined as a set of behavioral, cognitive, and emotional tendencies shaped by both biological and environmental factors, and characterized by relative stability as well as developmental plasticity (Corr and Matthews, 2020). Many personality theories argue that personality is hierarchical, in which certain traits exert more influence than others (Allport, 1927; Cattell, 1943; Tupes and Christal, 1992). Cattell’s theory of trait anxiety is rooted in observable behavior patterns (Corr and Matthews, 2020), whereas Eysenck, incorporating both biological and learning perspectives, proposed three core dimensions of personality: extraversion, neuroticism, and psychoticism. These traits, particularly neuroticism, have been empirically associated with increased vulnerability to anxiety symptoms. While personality traits can be shaped by life experiences such as trauma or illness, personal identity is often regarded as more enduring and resistant to situational change (Shoemaker and Tobia, 2019; Finlay and Starmans, 2022). This distinction highlights a key difference between the dynamic aspects of personality and the structural continuity of identity. In situations involving negative or threatening stimuli, individuals with a strong sense of personal identity may be better able to synthesize information and regulate emotional responses, thereby reducing the intensity of anxiety. In this sense, identity may serve a protective function.

In the present study, we propose that personality serves as a mediator between personal identity and trait anxiety. The formation of the self, predating personality development, and the emergence of self-synthesis mechanisms during early developmental stages are pivotal. While personality is often considered to reach maturity in young adulthood, identity is not always externally observable, whereas personality assessments typically rely on consistent behavioral expressions and social interactions. This conceptual distinction informs our hypothesis regarding the indirect pathway from identity to anxiety via personality.

In this expanded discussion, we further specify that personality is a multifaceted construct composed of distinct traits, each potentially exerting different effects on the identity–anxiety relationship. For instance, neuroticism is conceptually and empirically the most relevant trait in this context, as it is strongly associated with emotional instability and a heightened tendency to experience anxiety (McCrae and Costa, 1991). In contrast, extraversion is generally linked to resilience and positive affect, potentially serving as a protective factor. Meanwhile, psychoticism, which reflects detachment and impulsivity, may undermine both emotional integration and identity coherence. These distinctions are particularly relevant given our use of the Eysenck Personality Questionnaire (EPQ), which captures these three dimensions.

Empirical studies support these associations: neuroticism has been shown to predict both generalized and specific anxiety disorders (Kotov et al., 2010), while extraversion is negatively correlated with anxiety symptoms and positively related to meaning in life and self-concept clarity (Hassan et al., 2021; De Moor et al., 2022). By articulating the trait-specific roles of personality, our model gains both theoretical precision and empirical relevance, enhancing our understanding of how identity indirectly impacts anxiety.

### 1.3 Meaning in life as a mediator between identity and anxiety

A substantial body of research has demonstrated a strong correlation between meaning in life and overall mental health (Parra, 2020; Arslan et al., 2022; Costanza et al., 2019). Steger et al. argue that the meaning in life encompasses two factors: (1) search for the meaning in life (SML), representing the motivational aspect of meaning in life; (2) presence of meaning in life (PML) represents the cognitive aspect of meaning in life (Steger et al., 2006). Meaning in life is related to many factors such as emotions, social connections, religion and worldview, death, and self (King and Hicks, 2021). A large number of studies focus on the relationship between positive emotions such as the meaning in life and well-being; in recent years, some studies believe that meaning in life is one of the factors that can reduce anxiety, but the existing research is mostly concerned with the relationship between meaning in life and death anxiety, or focuses on special groups (Zhang et al., 2019; Liu et al., 2022). Few investigations have addressed how meaning in life relates to trait anxiety in non-clinical, general populations.

Coherence or comprehension—one of the cognitive dimensions of meaning in life—refers to the perception that life events are predictable and structured, enabling individuals to mentally simulate and anticipate future scenarios (George and Park, 2016; Heintzelman et al., 2013). However, much of the current research focuses on external coherence (e.g., coherence with environmental patterns), rather than inner psychological coherence, such as the continuity of the self over time—i.e., personal identity. Notably, research by Nichols et al. (2018) suggests that denying the persistence of personal identity can reduce fear of death (Nichols et al., 2018), implying an indirect connection between identity and one’s perceived meaning in life. Based on these theoretical connections, this study proposes that meaning in life mediates the relationship between personal identity and trait anxiety. A coherent and continuous sense of self may support one’s perceived meaning in life, which in turn may buffer against the experience of persistent anxiety.

### 1.4 Overview of the present research

The present study investigates the predictive role of personal identity in trait anxiety among Chinese college students. Drawing upon theoretical models of self-continuity and emotional regulation, we examine whether personality traits and the perceived meaning in life mediate this relationship.

Specifically, we propose and test the following hypotheses:

1. Personal identity is a significant predictor of trait anxiety;
2. Personality mediates the relationship between personal identity and trait anxiety;
3. Meaning in life mediates the relationship between personal identity and trait anxiety.

To address these hypotheses, we employed a combination of adaptive LASSO network analysis and Bayesian modeling to identify key pathways and conditional dependencies among variables.

This study contributes to the literature by offering an integrated model of anxiety that foregrounds identity-based mechanisms. It also provides empirical support for the development of psychological interventions that target self-coherence as a means of anxiety reduction in university student populations.

## 2 Method

### 2.1 Participants

Participants were undergraduate students recruited from universities in Wenzhou and Nanjing, China, using a simple random cluster sampling method. A total of 689 responses were collected via the online platform Questionnaire Star.

To ensure data quality, we applied several exclusion criteria: responses were excluded if participants provided implausible information (e.g., an age of 100 years or survey completion in an unrealistically short time), displayed uniform or repetitive answering patterns suggestive of disengagement, or scored 70 or above on the Lie Scale (EPQ-L) of the Eysenck Personality Questionnaire, which indicates a tendency toward socially desirable or exaggerated responding. These criteria were based on the questionnaire manual and established validation procedures.

After applying these criteria, 76 cases were excluded, resulting in a final sample of 613 valid responses. The mean age of participants was 20.42 years (SD = 1.46), ranging from 17 to 25. Detailed demographic information is presented in Table 1.

**Table 1:** Demographic information table.

Condition	Category	Frequency	Ratio
Age	Mean (SD)	20.42 (1.46)	
	Range	17–25	
Gender	Male	207	33.77%
	Female	406	66.23%
Education	Undergraduate	613	100.00%

### 2.2 Measures

#### 2.2.1 State–trait anxiety inventory

The questionnaire consists of 40 items and 2 sub-tables: Items 1–20 are the State Anxiety Scale (S-AI) and items 21–40 are titled Trait Anxiety Scale (T-AI). Responses were made on a 4-point Likert-type scale. The correlation coefficient between the initial test part and the retest part of the Chinese version of the questionnaire used in this study was 0.90. The correlation coefficient between the first test part and the retest part of S-AI is 0.88; the correlation coefficient between S-AI and T-AI is 0.84; the correlation coefficient between S-AI retest part and T-AI retest part is 0.77 (Spielberger et al., 1971; Zheng et al., 1993). The Cronbach’s *α* coefficient for this questionnaire in this study was 0.902.

#### 2.2.2 Self-identity scale

The SIS, developed by Ochse and Plug (1986), is a 19-item measure designed to assess whether an individual has successfully resolved the identity crisis described in Erikson’s theory. Items are scored on a 4-point scale, with higher scores indicating better resolution of identity crises (Ochse and Plug, 1986). While the SIS does not measure other dimensions of personal identity, such as social or cultural identity, it provides a targeted assessment of identity crisis resolution, which is a core aspect of personal identity development. In this study, we adopted the definition of personal identity as the enduring uniqueness of an individual over time (Numerical Identity), and the SIS aligns with this definition by focusing on the resolution of identity crises. The internal consistency coefficient of the Chinese version was 0.727 (Li and Lou, 2009). The Cronbach’s *α* coefficient for this questionnaire in this study was 0. 785.

#### 2.2.3 Eysenck personality questionnaire

The Chinese version of the questionnaire has a total of 88 items, which are scored using a dichotomous method, and the norm is divided according to gender. The questionnaire consists of four subscales: Extraversion/Introversion (E), Neuroticism/Stability (N), Psychoticism/Socialization (P), and validity scale (L). Professor Gong’s revised Chinese version has a good reputation among his Chinese counterparts, but for the adult version he calculated coincidence indicators rather than reliability, and the coincidence rate between the items in the version and those in the original questionnaire was 87.5 to 97.82% (Gong, 1984). In this study, the Cronbach’s α coefficient of the E dimension was 0.788, the N dimension was 0.872, the P dimension was 0.697, and the L dimension was 0.682.

#### 2.2.4 The meaning in life questionnaire

The Meaning in Life Questionnaire (MLQ) contains 10 items, including two dimensions: The search for meaning in life (SML) and the presence of meaning in life (PML). The questionnaire uses a 7-point scale, with higher scores indicating a stronger sense in meaning in life (Steger et al., 2006). The total Cronbach’s α coefficient of the Chinese version was 0.71 (Liu and Gan, 2010). The Cronbach’s α coefficient of this questionnaire in this study was 0.833.

### 2.3 Procedures

The questionnaires were administered in December 2022 during the university examination period. Data collection was conducted in designated classrooms or individual settings, using a unified set of questionnaires distributed through the Questionnaire Star online platform. All testing sessions were supervised by psychology students who had received standardized training. Prior to completing the questionnaires, participants were informed of the voluntary nature of the study, the confidentiality of their responses, and the importance of answering honestly. Written informed consent was obtained from both participants and class instructors. Each session took approximately 10 min to complete. All data collection procedures were conducted in accordance with ethical standards and institutional guidelines.

### 2.4 Data analysis

JASP 0.16.2.0, R language 4.1.3 and R studio 4.1.3 were used for data analysis and network analysis. Bayesian network models were constructed using GeNIe 3.0 Academic.

The following R packages were used for data analysis:

- **bootnet**: For network analysis and visualization.
- **mgm**: For estimating network models and calculating predictability.
- **qgraph**: For visualizing network graphs.
- **NetworkTools**: For centrality analysis and network diagnostics.
- **dplyr**: For data manipulation and preprocessing.

Network analysis is a recently emerged technique with broad applications. The network can investigate the relative strength of various factors, with the thickness of the lines representing the difference in weight, and the thicker the line, the stronger the correlation (Epskamp et al., 2018). The Adaptive Lasso Network Analysis used in this study is a method that combines the adaptive Lasso technique with network analysis. Its partial correlation coefficient describes the degree of association between two nodes (variables) while accounting for the influence of other nodes. The partial correlation coefficient helps eliminate the effects of other nodes, thus more accurately assessing the direct relationship between two nodes. Adaptive Lasso Network Analysis assists in selecting the most relevant features or nodes within complex networks. The penalty parameter (lambda) for the adaptive Lasso was selected using a 10-fold cross-validation procedure, which optimizes the model by minimizing prediction error and selecting the best lambda value to prevent overfitting and enhance generalizability.

The Bayesian model is a statistical model is utilized to describe uncertainty through probability modeling. Its core concept involves combining prior knowledge (existing information about parameters) with observed data to infer the posterior probability distribution of parameters. Bayesian network models are probabilistic graphical models that represent a set of variables and their conditional dependencies through directed acyclic graphs (DAGs). Bayesian networks are well suited for predicting events that occur and predicting the likelihood that any one of several possible known causes is a contributing factor (Ben-Gal, 2008).

The high and low groups for each node were created using the Uniform Counts discretization method in GeNIe3.0. This method divides the data into groups with an equal number of observations, ensuring balanced group sizes and minimizing bias in the analysis. Specifically, the data were divided into two groups (high and low) based on the median value of each variable.

## 3 Results

### 3.1 Descriptive statistics

Table 2 presents the descriptive statistics for all questionnaire measures, including means and standard deviations, based on the full sample of 613 participants.

**Table 2:** Descriptive statistics for questionnaire measures (*N* = 613).

Measure	Mean	SD
EPQ-P	51	11.84
EPQ-E	52.76	10.02
EPQ-N	49.21	13.19
EPQ-L	44.01	9.4
PML	26.44	5.18
SML	23.36	5.83
SIS	51.01	6.1
S-AI	43.21	10.47
T-AI	44.56	9.73

### 3.2 Correlation analysis

**Figure 1:**
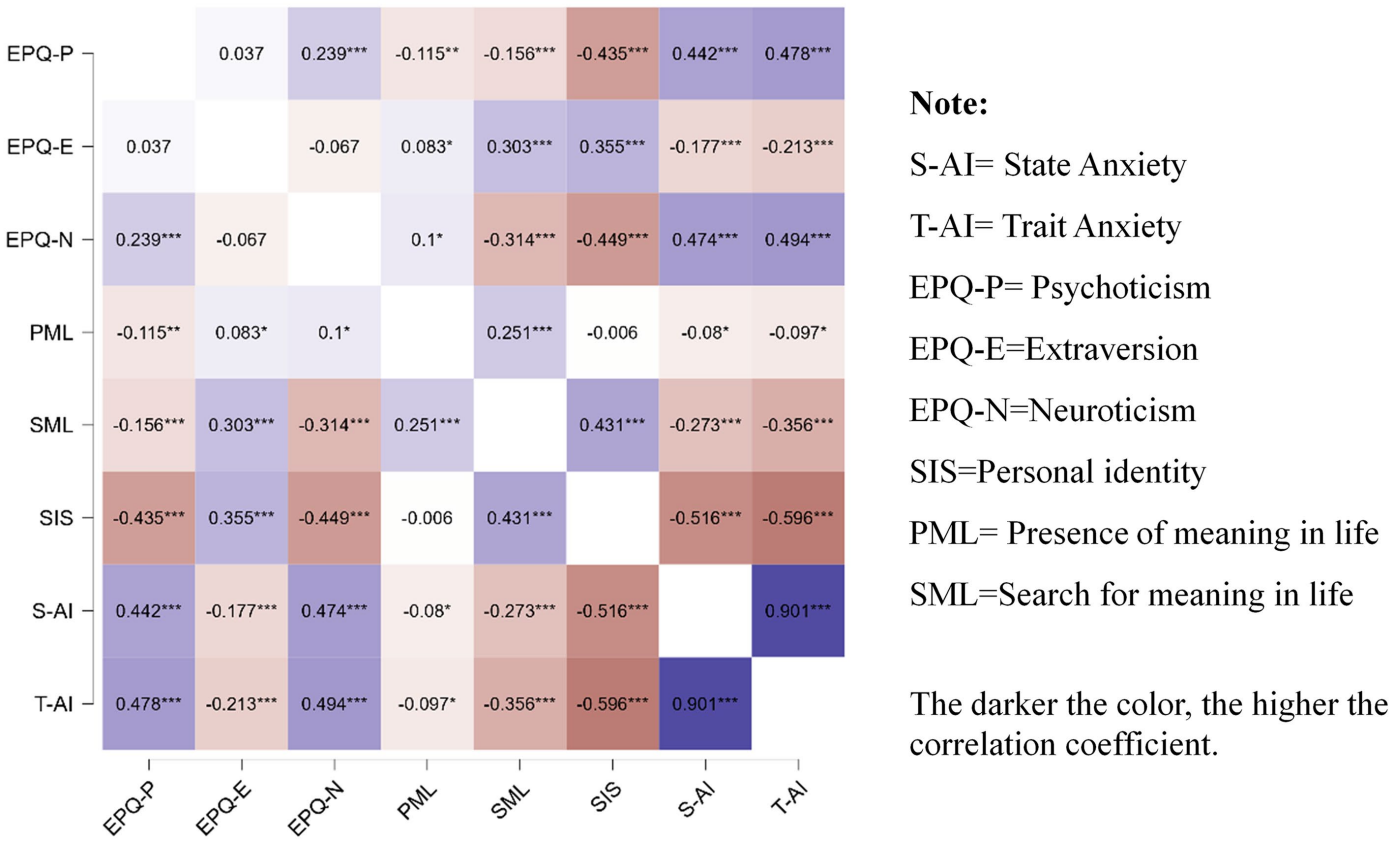
Heat map of correlation between variables (*n* = 613).

Pearson correlation coefficients were calculated to examine the relationships among personal identity, trait anxiety, personality traits, and meaning in life. A heatmap was generated to visualize the correlation matrix. The results showed that personal identity was significantly positively correlated with extraversion and the search for meaning in life, and significantly negatively correlated with state anxiety, trait anxiety, psychoticism, and neuroticism. State anxiety and trait anxiety were strongly positively correlated with each other. Both were significantly negatively correlated with extraversion, the search for meaning in life, and the presence of meaning in life, and positively correlated with psychoticism and neuroticism (see Figure 1).

### 3.3 Network analysis

**Figure 2:**
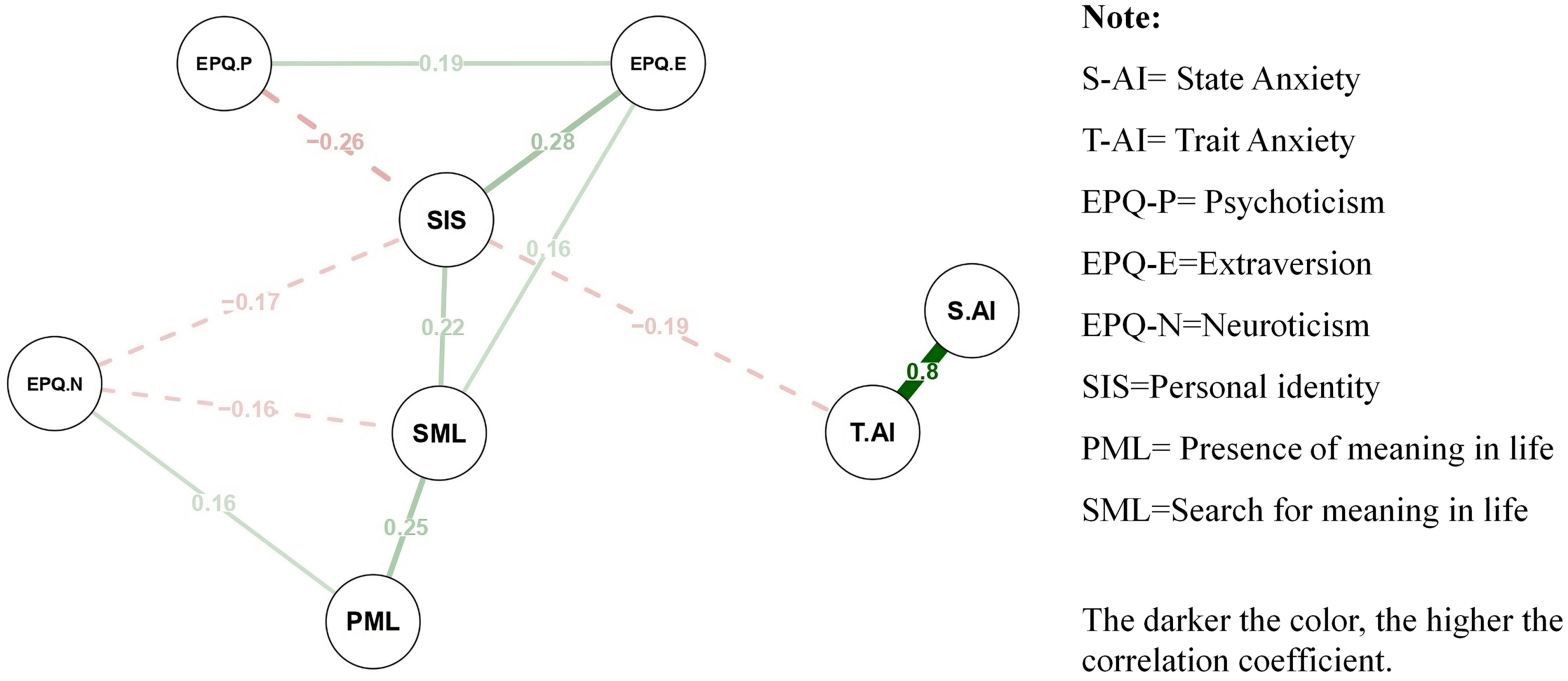
Adaptive LASSO network (*n* = 613).

Adaptive LASSO network analysis was conducted using the bootnet package to estimate the network structure of the variables. The thickness of the edges in the network represents the strength of the partial correlations between variables, with green edges indicating positive correlations and red edges indicating negative correlations (see Figure 2).

mgm package and other calculations were used to determine the predictability of each node. The predictability of each node refers to, in network analysis, the ability to predict the value of each node (variable) in the network using information from other nodes (see Table 3).

**Table 3:** Predictability of each node.

No.	Variable	RMSE	*R*^2^
1	EPQ.P	0.822	0.323
2	EPQ.E	0.883	0.220
3	EPQ.N	0.812	0.340
4	PML	0.928	0.137
5	SML	0.827	0.315
6	SIS	0.691	0.522
7	S.AI	0.427	0.817
8	T.AI	0.395	0.844

**Figure 3:**
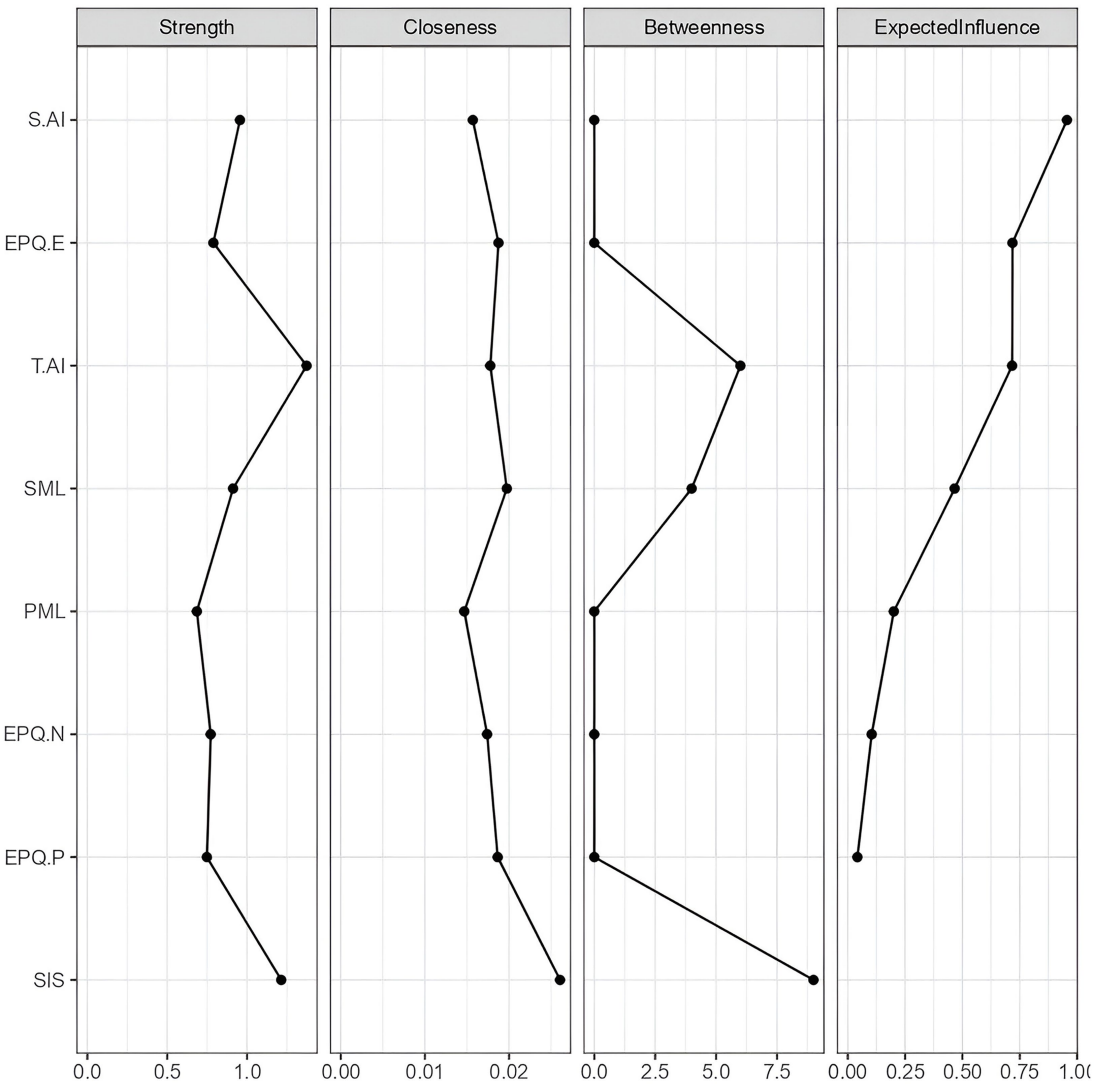
Centrality indices.

To further examine the factors affecting node predictability, this study calculated centrality indices, which measure the importance or centrality of nodes in a network. Specifically, strength, closeness centrality, and betweenness centrality were computed to evaluate the significance of each variable in the network (see Figure 3). The results show that SIS scores high on all three measures: strength, closeness centrality, and betweenness centrality (see Figure 3).

Subsequently, network validity was assessed using the bootnet package, and the results were presented in terms of correlation stability coefficients (CS-coefficients). The CS coefficient should not fall below 0.25 and is preferably higher than 0.50 (Epskamp et al., 2018). The stability analysis revealed that both betweenness centrality (CS = 0.594) and closeness centrality (CS = 0.594) exceeded the critical threshold of 0.5. Additionally, both strength (CS = 0.750) and expected influence (CS = 0.750) surpassed 0.75.

**Figure 4:**
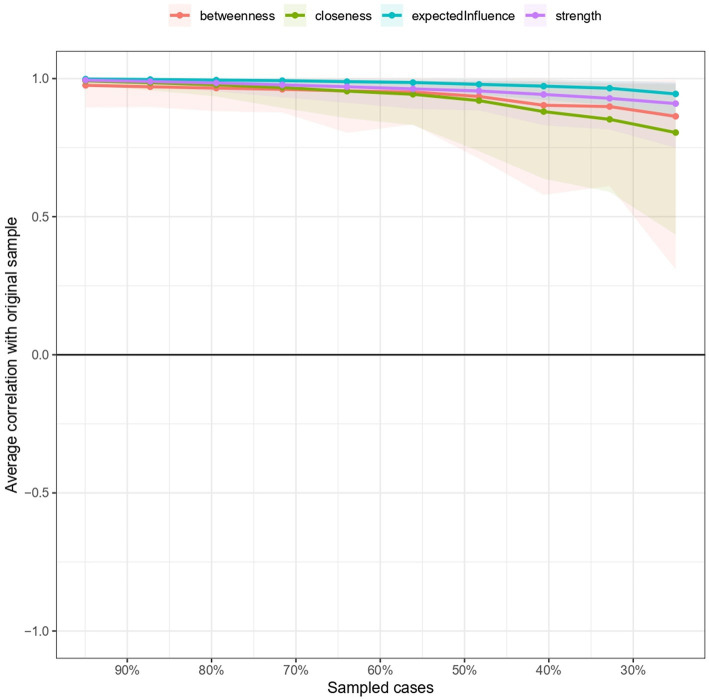
Bootstrapped stability coefficients of centrality indices.

We also calculated correlation stability coefficients (CS-coefficients) for the centrality indices as a means of quantifying stability and interpretability (Epskamp and Fried, 2018). To achieve this, we conducted a “case-dropping subset bootstrap” using the bootnet package in R [48]. The outcome of this analysis, as presented in Figure 4. The figure depicts the maximum proportion of the original sample that can be dropped (X axis) while confidently retaining a centrality (expected influence or strength) correlation above 0.7 with the original sample (Y axis). The solid lines indicate the centrality correlations. The shaded areas indicate the 95% confidence interval for the centrality correlations. Centrality correlations greater than 0.7 indicate that centrality was estimated similarly in the full sample and in smaller subsets of the original sample. All the indices were estimated with adequate accuracy, meaning that the network can be meaningfully interpreted.

### 3.4 Bayesian network

#### 3.4.1 Construct Bayesian network model

Bayesian network models were constructed using GeNIe 3.0 Academic to explore the causal relationships between variables. The model was trained using 306 randomly selected data points, and sensitivity analysis was performed to identify the most influential variables. The accuracy of the model was validated using the remaining 307 data points.

#### 3.4.2 Learn parameters

The Bayesian model of trait anxiety prediction was constructed through GeNIe 3.0 Academic (64 bit). Three hundred and six data were randomly selected for discretization into two groups of high and low score, and parameter learning was carried out to determine the probability between each node. The model needs to learn the parameters several times to ensure the relative stability.

#### 3.4.3 Sensitivity analysis

Sensitivity analysis can be used to identify the factors that have the greatest influence on the target node in the Bayesian network model. This is done by investigating the effect of small changes in numerical parameters on the output parameters. Highly sensitive parameters affect the reasoning results more significantly. In this study, redundancy was removed by means of adaptive LASSO network analysis, and only SIS was retained as the parent node, but its sensitivity was still calculated. The average sensitivity of SIS was 0.229 (see Table 4).

**Table 4:** Bayesian model sensitivity analysis.

Group	SIS-low group	SIS-high group
T-AI-low group	0.23832999	0.69590643
T-AI-high group	0.76167001	0.30409357

S-AI = state anxiety; T-AI = trait anxiety; SIS = personal identity.

**Figure 5:**
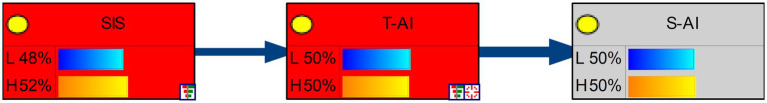
Bayesian network model.

The Bayesian model is as follows (see Figure 5).

#### 3.4.4 Model validity verification

Three hundred and seven data were used to verify the accuracy of the prediction probability of the model. The overall prediction accuracy of the model was 88.60, 87.25% for the group with high trait anxiety and 89.87% for the group with low trait anxiety (see Table 5).

**Table 5:** Accuracy of predicting probabilities of trait anxiety.

Group	Accuracy
Total	0.885993 (272/307)
T-AI-high group	0.872483 (130/149)
T-AI-low group	0.898734 (142/158)

S-AI = State Anxiety, T-AI = Trait Anxiety; EPQ.E = Extraversion; SIS=Personal identity.

## 4 Discussion

This study aimed to explore the predictive role of personal identity in trait anxiety and to examine whether personality traits and meaning in life act as mediators. Consistent with our expectations, the findings highlight personal identity as a central construct within the anxiety-related psychological network, both in terms of its structural positioning and predictive power. While the mediating roles of personality and meaning in life were not statistically supported in the final model, both constructs showed meaningful associations with identity and anxiety. These results invite a deeper examination of the ways in which a coherent sense of self may indirectly shape emotional vulnerability through personality configurations and existential orientation.

Adaptive LASSO network analysis identified personal identity as the key factor influencing trait anxiety levels. Furthermore, Bayesian network analysis suggested that personal identity can effectively predict trait anxiety, with a classification accuracy of 88.59%, indicating a robust predictive relationship between the two variables.

The relationship between anxiety and personal identity has been supported in previous studies, with personal identity frequently identified as a significant predictor of anxiety levels (Marcia, 1976; Hayward et al., 2020). Our findings reinforce this connection, suggesting that a coherent and stable personal identity may play a protective role against anxiety. Personal identity is often understood in terms of the “self,” a concept that encompasses both subjective awareness and the reflective understanding of oneself as an object (Olson, 2022; Drummond, 2020). This dual nature can be traced back to Kant’s distinction between transcendental and empirical self-consciousness, a framework which continues to influence psychological models of the self (Solomon, 1972; Longuenesse, 2013). Building on this, we interpret personal identity as both a functional process of integrating experiences (the “self-as-process”) and as the awareness of coherence across time (the “self-as-object”) (Burke, 2020).

Recent theories of predictive coding suggest that the brain continuously generates internal models to anticipate and interpret external stimuli (Clark, 2013). This aligns with our view that anxiety may emerge when the internal predictive model fails to match reality—a process we describe as a self-synthesis failure. Specifically, two conditions may give rise to anxiety: (1) the empirical model constructed by the self is inaccurate or inadequate, resulting in persistent mismatch; or (2) the individual’s tolerance for prediction errors is low, causing emotional overreaction to small discrepancies (Millidge et al., 2021). This interpretation also resonates with psychoanalytic theories, including Freud’s model of internal conflict between the id, ego, and superego, and Kohut’s Self Psychology, which frames anxiety as a consequence of fragmentation within the self. Across these perspectives, anxiety is consistently framed as a breakdown in internal integration—whether cognitive or emotional—and our findings provide empirical support for this conceptualization.

The types of predictive models constructed by the self—and the degree to which individuals tolerate mismatches between internal models and external reality—may be shaped by early interpersonal experiences. This aligns with attachment theory, which posits that early caregiving experiences shape internal working models that influence emotional regulation and threat sensitivity throughout life (Bowlby, 1969; Mikulincer and Shaver, 2007). These early-formed expectations not only govern interpersonal behavior but may also serve as templates for broader experiential processing, thereby modulating one’s capacity to maintain identity coherence under stress. Individuals with different attachment styles exhibit distinct relational patterns that serve as prototypes not only for interpersonal interactions but also for broader engagement with environmental stressors. These early-formed expectations can influence the flexibility or rigidity of one’s internal models, potentially affecting susceptibility to anxiety when faced with ambiguous or unpredictable situations.

The hypothesis that personality would serve as a partial mediator between personal identity and trait anxiety was not supported by the results of this study. One possible explanation is that network analysis, unlike traditional regression-based mediation approaches, is designed to eliminate redundant paths and highlight the most direct associations between variables. As a result, the indirect effects observed in previous studies may not emerge as significant once overlapping influences are removed. However, it is worth noting that while the overall mediating effect of personality was not statistically significant, trait-level analysis still revealed meaningful trends. For example, neuroticism showed a strong positive correlation with trait anxiety, consistent with its established role in emotional vulnerability (McCrae and Costa, 1991). Conversely, extraversion was negatively associated with anxiety, suggesting a potential protective function. Psychoticism, although less studied in this context, was positively associated with identity instability and anxiety. These patterns, though not meeting mediation criteria in our model, point to the heterogeneous functions of individual traits and highlight the need for further research using dimensional or longitudinal approaches. This nuanced understanding aligns with recent personality–psychopathology frameworks that emphasize trait-specific pathways over generalized constructs (Kotov et al., 2010).

This study found that search for meaning in life was associated with neuroticism and extraversion, and although it did not directly affect anxiety level, we still consider it a factor of concern. Meaning in life is a concept full of existentialism, Frankl and Heidegger both explored the importance of meaning in life (Shim, 2020; Frankl, 1946/2010; Heidegger, 1927/2012). These philosophical references serve to contextualize the construct of meaning in life within existential psychology, and are complemented by empirical psychological literature (e.g., George and Park, 2016; King and Hicks, 2021). Although the search for meaning in life did not show a direct effect on anxiety in our model, it was significantly associated with both extraversion and neuroticism, suggesting its relevance as a psychological construct within the broader personality-emotion framework. The lack of a direct effect from meaning in life to anxiety may reflect indirect or moderated pathways not captured by the current model. For instance, meaning in life may exert its influence through emotion regulation, resilience, or value orientation, acting as a higher-order protective factor (George and Park, 2016). Additionally, our use of network analysis may have filtered out redundant paths, especially in the presence of strong mediators like personality or identity. Another possible reason is construct overlap: meaning in life and identity coherence may share variance, reducing their independent predictive value when modeled simultaneously. This pattern aligns with previous research showing that individuals with higher extraversion or lower neuroticism tend to report greater meaning in life (King and Hicks, 2021). As meaning in life is often connected with existential orientation and emotional resilience, its indirect role in anxiety regulation may warrant further exploration, even if it was not statistically significant in the current network structure.

### 4.1 Theoretical and practical implications

This study suggests that personal identity exerts a predictive influence on anxiety levels in college students. This has important theoretical and practical implications, particularly in the context of psychological intervention. Enhancing one’s sense of identity and reinforcing self-continuity may help mitigate anxiety by improving internal coherence and reducing the mismatch between perceived experience and internal expectations. For example, in cases of test anxiety—which may persist even after the completion of low-stakes exams—conventional interventions such as exam preparation or stress-coping strategies may be insufficient. In such instances, interventions aimed at reorganizing one’s internal narrative or experiential model may be necessary. This could involve guiding individuals to reframe their life experiences in ways that strengthen their identity and increase resilience to uncertainty.

Attachment theory offers additional insights into how identity-based interventions might be structured. The concept of a “secure base,” originally proposed by Bowlby and Ainsworth, suggests that individuals require a sense of psychological safety to explore new experiences. Interventions that attempt to modify one’s self-concept or meaning structure may temporarily disrupt self-continuity; therefore, it is important to establish a stable and supportive therapeutic context to ensure that individuals feel safe enough to engage in this cognitive reorganization.

Furthermore, our findings regarding meaning in life—despite the lack of direct effect on anxiety—suggest that it may serve as a complementary pathway for intervention. Meaning in life is deeply rooted in existential theory, as explored by Frankl and Heidegger, and has been shown to contribute to emotional well-being. Studies have found that exposure to structured and predictable daily stimuli can enhance one’s sense of meaning, thereby offering a subtle yet effective route for anxiety reduction (Heintzelman et al., 2013; Heintzelman and King, 2013, 2014; King and Hicks, 2021). Encouraging individuals to engage in meaningful routines and redefine their personal goals may thus serve as an accessible and non-invasive therapeutic tool.

### 4.2 Limitations and future directions

The present study adopted a cross-sectional design, which is effective for identifying structural associations among variables such as personal identity, trait anxiety, personality traits, and meaning in life. While this design allows for efficient hypothesis testing, it does not support strong causal inference. To partially address this limitation, we employed a two-stage analytic strategy combining adaptive LASSO network modeling with Bayesian analysis to explore directional patterns based on probabilistic structures. However, causal conclusions remain tentative and should be verified through longitudinal or experimental research.

Several additional limitations should be acknowledged. First, reliance on self-report instruments may introduce bias, including social desirability effects, recall errors, or response distortions. Future studies could benefit from incorporating multiple sources of data—such as clinical interviews, behavioral tasks, or physiological measures—to enhance the validity and reliability of findings.

Second, the sample consisted solely of Chinese university students, which may limit the generalizability of results across cultures and age groups. Cultural factors, such as individualism–collectivism orientations, may shape both identity development and its relationship with anxiety. For example, personal identity in Western contexts often emphasizes autonomy, whereas Eastern contexts may prioritize relational harmony. Similarly, developmental stage may moderate the observed associations, and future research should include more diverse populations, including adolescents, working adults, older individuals, and clinical samples.

Finally, further work is needed to examine additional variables that might moderate or mediate the identity–anxiety relationship, such as emotional regulation, attachment style, or value orientation. Longitudinal and ecological designs could be particularly valuable in capturing the dynamic and context-sensitive nature of identity development and its psychological consequences.

## 5 Conclusion

This study examined the predictive role of personal identity in trait anxiety among Chinese university students, with attention to the potential mediating effects of personality traits and meaning in life. Using adaptive LASSO network analysis and Bayesian modeling, we found that personal identity plays a central role in the anxiety-related psychological network and demonstrates strong predictive accuracy for individual differences in trait anxiety.

Although our hypotheses regarding the mediating effects of personality and meaning in life were not statistically supported in the final model, the findings nonetheless suggest that a stable and coherent sense of self is a key protective factor against anxiety. These results contribute to a growing body of research that frames anxiety as a consequence of disrupted internal integration, or failures in self-coherence.

Importantly, our findings underscore the need to address personal identity in both theoretical models and practical interventions targeting anxiety. Identity-based interventions may offer a promising route for enhancing emotional resilience in university populations, particularly in contexts characterized by developmental uncertainty and self-exploration.

## Data Availability

The original contributions presented in the study are included in the article/supplementary material, further inquiries can be directed to the corresponding author/s.
